# Characteristics and Clinical Significance of T-Cell Receptor Repertoire in Hepatocellular Carcinoma

**DOI:** 10.3389/fimmu.2022.847263

**Published:** 2022-03-16

**Authors:** Zifei Wang, Yu Zhong, Zefan Zhang, Kaiqian Zhou, Zhihao Huang, Hao Yu, Longqi Liu, Shiping Liu, Huanming Yang, Jian Zhou, Jia Fan, Liang Wu, Yunfan Sun

**Affiliations:** ^1^ College of Life Sciences, University of Chinese Academy of Sciences, Beijing, China; ^2^ Beijing Genomics Institute at Shenzhen, Shenzhen, China; ^3^ Zhong-Hua Precision Medical Center, Zhongshan Hospital, Fudan University-BGI, Shanghai, China; ^4^ Department of Liver Surgery & Transplantation, Liver Cancer Institute, Zhongshan Hospital, Fudan University, Key Laboratory of Carcinogenesis and Cancer Invasion, Ministry of Education, Shanghai, China; ^5^ Shenzhen Key Laboratory of Single-Cell Omics, BGI-Shenzhen, Shenzhen, China

**Keywords:** hepatocellular carcinoma, biomarker, diagnosis, T-cell repertoire, complementarity-determining region 3

## Abstract

Several studies have demonstrated that the T-cell receptor (TCR) repertoire is associated with prognosis and immune therapy response in several types of cancer. However, the comprehensive features of TCR repertoire in tumor-infiltrating and circulating T cells, as well as its clinical significance of diagnosis in hepatocellular carcinoma (HCC) patients, are still unknown. In this study, we perform paired tumor/peritumoral tissues and peripheral blood samples from 58 patients with HCC and sequenced them with high-throughput TCR to comprehensively analyze the characteristics of TCR and the clinical significance of peripheral TCR sequence. By exploring the abundance and diversity of TCR repertoires, we observe that there was a significantly higher TCR diversity in peripheral blood than in tumoral and peritumoral tissues, while tumoral and peritumoral tissues showed similar TCR diversity. A substantial difference in the usage frequencies of several Vβ, Jβ genes, and TCRβ VJ pairings was found among three types of tissues. Moreover, we reveal that HCC patients have a unique profile of TCR repertoire in peripheral blood in contrast to healthy individuals. We further establish an HCC diagnostic model based on TCRβ VJ pairing usage in peripheral blood, which yields a best-fit area under the curve (AUC) of 0.9746 ± 0.0481 (sensitivity = 0.9675 ± 0.0603, specificity = 0.9998 ± 0.0007, average of 100 repeats) in the test set. Our study describes the characteristics of tissue infiltration and circulating T-cell bank in patients with HCC and shows the potential of using circulating TCR sequence as a biomarker for the non-invasive diagnosis of patients with HCC.

## Introduction

Liver cancer is the seventh most frequently diagnosed cancer and the third cause of cancer death worldwide, accounting for 905,677 new cases and 830,180 deaths in 2020 (1, 2). Occupying 75%–90% of cases, hepatocellular carcinoma (HCC) represents the most common type of liver cancer (3, 4). Surgical resection remains one of the most effective curative treatments (5). However, up to 70% of patients may relapse within 5 years after curative surgery (6). Moreover, there are limited therapeutic options for patients with advanced-stage HCC, their general survival time is only 6 months (7). The high recurrent rate and poor survival are major obstacles to improving the clinical outcome of HCC patients (8–11). Although several molecular markers, such as Glypican-3 (GPC3), Des-γ-carboxyprothrombin (DCP), and Osteopontin (OPN), have been reported as potential diagnostic or prognostic markers of HCC, none has translated into routine clinical practice because of their limited accuracy in predicting HCC progression (12). As for alpha-fetoprotein (AFP), it is the most widely used non-invasive biomarker for HCC diagnosis, and there are still about 30% to 40% of HCC patients who are AFP negative (13, 14). Therefore, identifying more accurate biomarkers to facilitate early diagnosis or monitoring the prognosis of HCC is an urgent need (15).

Previous studies revealed that T-cell immunity is closely related to tumor development and clinical outcomes in different types of tumors, including gastric cancer, primary testicular lymphoma, and melanoma (16–18). T cells recognize neoantigens presented on tumor cells using their T-cell receptors (TCRs). The subsequent massive expansion of T cells indicates the selective activation of T cells according to specific recognition of tumor neoantigens (19, 20). The TCR repertoire co-evolutes with neoantigens over time, which suggests that the specific TCR repertoire can reflect the immune response and characteristics of disease progression (21). With the use of high-throughput sequencing of the TCR repertoire, a series of studies have reported that the TCR repertoire can be a prognostic factor for some cancers such as cervical cancer, nasopharyngeal carcinoma, and renal cell carcinoma (22–24). As for liver-associated diseases, TCR repertoire signatures of three chronic liver diseases, including primary biliary cirrhosis (PBC), primary sclerosing cholangitis (PSC), and alcoholic liver disease (ALD), are different, suggesting the existence of disease-associated antigenic repertoires (25). The existence of different expression profiling of TCRβ complementarity-determining region 3 (CDR3) among healthy adults, hepatitis B virus (HBV) patients, and liver cancer patients at the tissue level have also been demonstrated (26). Moreover, for HBV-associated HCC, a study observed a weak TCR repertoire similarity between tumor and matched non-tumor tissues (27). Another study found that the similarity of TCR repertoires between tumor and paired peritumoral tissues could serve as efficient prognostic indicators when combined with the TNM staging (28). However, a comprehensive landscape of TCR repertoires in infiltrating and circulating T cells of HCC patients still needs to be elucidated when evaluating the peripheral TCR repertoire as a potential diagnostic or prognostic biomarker of HCC.

In this study, from 58 HCC patients using high-throughput TCR sequencing, we elucidate the TCR repertoire in the tumor, in tumor-adjacent non-tumor tissues, and peripheral blood. We found that the TCRβ CDR3 diversity in peripheral blood was higher than those in the other two tissues. We made a detailed inter-tissue comparison of these TCR repertoires and then demonstrated a heterogeneous usage pattern of TCRβ variable (V) and joining (J) genes among these three tissues. Interestingly, we observed a unique profile of TCR repertoire in the blood from HCC patients when compared with that of healthy donors. Through the least absolute shrinkage and selection operator (LASSO) regression model, we prove that the peripheral TCRβ VJ pairing can distinguish HCC patients. In summary, our data reveal the potential clinical value of the TCR repertoire in peripheral blood for the diagnosis of HCC.

## Materials and Methods

### Patient Characteristics

Patients were enrolled from January 2015 to June 2017 at the Zhongshan Hospital Fudan University (Shanghai, China). HCC was diagnosed and treated according to the American Association for the Study of Liver Diseases guidelines (5). Inclusion criteria were 1) definitive pathological diagnosis of HCC based on the WHO criteria; 2) curative resection, defined as complete macroscopic removal of the tumor (29); 3) no prior anticancer treatment, including chemotherapy, radiotherapy, targeted therapy, or immune therapy; and 4) no history of other types of malignancies. A total of 58 eligible patients were recruited into this study. Tumor samples, adjacent non-tumor tissue samples, or peripheral blood samples were collected for high-throughput TCR sequencing. Patients’ liver function was evaluated using the Child–Pugh score and albumin–bilirubin (ALBI) score (30). The tumor stage was determined according to the Barcelona Clinic Liver Cancer (BCLC) and China Liver Cancer (CNLC) staging system (31, 32). All peripheral blood samples were obtained before surgery, and all surgically resected tissue samples were confirmed independently by 2 experienced pathologists.

All patients were prospectively monitored by liver function test, serum AFP, and abdominal ultrasonography at each follow-up visit. MRI or CT of the abdomen was performed every 6 months. If intrahepatic recurrence or distal metastasis was clinically suspected on the basis of symptoms or unexplained elevation of tumor marker levels, MRI, CT, or bone scan was performed immediately. Every patient was followed up every 2 to 3 months in the first 2 years after surgery and then every 3 to 6 months thereafter. Overtime survival was the interval from the date of surgery to either the date of death or the last follow‐up visit. Time to recurrence (TTR) was defined as the interval between the date of surgery and the date when intrahepatic recurrence or extrahepatic metastasis was first diagnosed (33).

The study protocol was approved by the Institutional Ethics Committee of the Zhongshan Hospital, Fudan University, with approval number B2019-060(2) and was conducted according to the ethics guidelines of the 1975 Declaration of Helsinki. Written informed consent was granted by each of the recruited patients.

### DNA Isolation and T-Cell Receptor Sequencing

The rearranged TCR repertoires were amplified by the multiplex PCR (MPCR) method described in previously published papers (34). In short, genomic DNA for each sample was extracted using a Genomic DNA Extraction Kit (TIANGEN, Beijing, China, Cat# DP304-03#) and then amplified using the Multiplex PCR Kit (QIAGEN, Valencia, CA, USA, Cat# 206143#) with 32 forward V primers and 13 reverse J primers. The cycling conditions were as follows: 95°C for 15 min, 30 cycles at 94°C for 30 s, 60°C for 90 s, and 72°C for 30 s, plus a final extension at 72°C for 5 min. Before the beginning of the study, the synthesized various TCRβ templates were used to evaluate the PCR bias of multiple primers and then supported us with the optimized primer mix to minimize the PCR bias (35). At last, the PCR product was purified by running on a 2% agarose gel reaction, and then paired-end 100-bp reads were outputted on the BGISEQ-500 sequencer.

### Immune Repertoire Analysis

IMonitor (36) pipeline was used to analyze TCR sequencing data. First, low-quality reads were discarded, and clean paired-end reads were merged by their overlapping nucleotides. Second, the merge reads were assigned to VDJ germline segments and alleles from IMGT (http://www.imgt.org/) database by BLAST alignment and further re-alignment. Third, the sequencing error was corrected. Then the abundant and Shannon’s entropy of each TCR VDJ segment was calculated.

### Principal Component Analysis

Principal component analysis (PCA) of peripheral blood from 439 healthy donors (37) and 43 HCC patients was performed by the scaled abundance of overlapped 624 TCRβ VJ pairings. The prcomp () function in R was used to performed PCA, and principal component (PC) 1 and PC2 were used for visualization.

### Identification of Discriminative TCRβ VJ Pairings

Discriminative TCRβ VJ pairings between peripheral blood from healthy donors (37) and 43 HCC patients were identified using the “FindAllMarkers” function, implemented in the Seurat package; discriminative TCRβ VJ pairings with log-scaled fold change ≥0.25 and area under the curve (AUC) >0.7 between two groups of samples were identified using the receiver operating characteristic (ROC) test.

### Absolute Shrinkage and Selection Operator Logistic Regression

LASSO regression analysis was performed by glmnet package to construct a model to predict HCC patients and healthy people based on TCRβ VJ pairing usage from peripheral blood. By using random partitioning of the dataset, the 80% samples of 43 HCC (35/43) were randomly selected and set as the training group, and the rest of the 20% (8/43) of samples were used as the test group. In the training group, the number of HCC samples and normal controls (37) was sampled equally to build the classification model. Finally, 35 samples were randomly selected from both HCC patients and healthy people as the training group (70 samples). And the rest of the samples were selected from both HCC patients and healthy people as the testing group (412 samples). The response type of model output is set “binomial.” The lambda that gives minimum mean cross-validated error was determined by the “cv.glmnet” function. Given the lambda, the selected features and coefficient of these features were determined. Then the prediction result was calculated by the regression model using the “predict” function. The AUC was calculated by the ROC R package. To exclude just a chance occurrence and demonstrate the robustness of the LASSO model, we repeated the above process 100 times. The model coefficients for features, accuracy, recall sensitivity, precision, and specificity from 100 repeats are recorded.

### Statistical Analysis

Paired t-test was used to compare the abundance and diversity of TCRβ CDR3 clonotypes among three tissues. The comparisons of multiple groups and the abundance of TCRβ VJ among tumors, adjacent normal tissues, and peripheral blood were performed using the paired Kruskal–Wallis test. The unpaired Wilcoxon test was used to compare the proportion of shared clones of each two tissues, while the paired Wilcoxon test was used to compare the proportion of shared clones/clonotypes among three tissues. The *p*-values of multiple comparisons were adjusted by the Bonferroni correction. A chi-square test was used to test the association between these clonotypes with patient clinical parameters. All statistical analyses and presentations were performed using R.

## Results

### Elucidation of T-Cell Receptor Repertoires in Hepatocellular Carcinoma Patients

**Figure 1:**
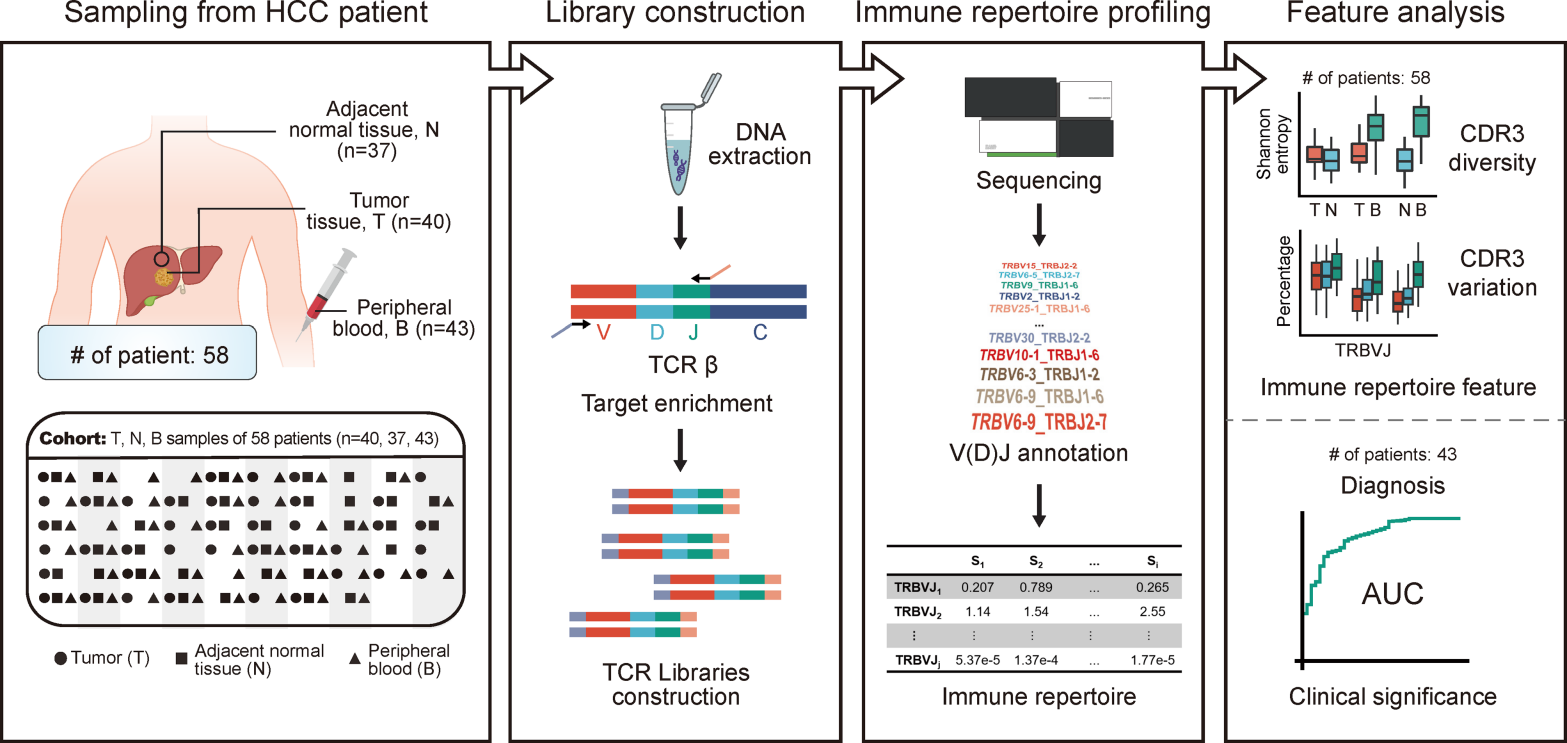
The design and flowchart of this study. HCC cohort: 40 tumors, 37 adjacent non-tumor tissues, and 43 peripheral blood samples were collected from 58 patients diagnosed with HCC. The TCR repertoire of patients was profiled using high-throughput TCR sequencing. The abundance and diversity of TCR were characterized based on HCC cohort, while the clinical significance of circulating TCR repertoire for diagnosis and prognosis was explored based on peripheral blood samples from HCC cohort. HCC, hepatocellular carcinoma; TCR, T-cell receptor.

To explore the heterogeneity of TCR features among different tissue types in HCC patients, we performed TCR sequencing of 40 tumor samples (T), 37 adjacent non-tumor tissue samples (N), and 43 peripheral blood samples (B) collected from 58 patients diagnosed with HCC. Some of the 58 patients collected only one tissue of T, N, and B; some patients collected two of them, while some patients collected three of them. The specific tissues collection strategy is shown in **Figure 1** and **Supplementary Table 1**. The baseline characteristics of the HCC cohort are shown in **Table 1**. For a total of 120 samples from 58 patients, we acquired an average of 11,037,670 ± 5,581,145 clean sequencing reads, 47,565 ± 25,114 distinct CDR3 nucleotide (nt) clonotypes, and 30,246 ± 15,807 distinct CDR3 amino acid (aa) clonotypes (**Supplementary Figure 1**). For each sample, a total of 624 VJ gene combinations were identified, including 48 distinct Vβ gene segments and 13 distinct Jβ gene segments. The detailed sequencing data information is shown in **Supplementary Table 1**.

**Table 1:** The baseline characteristics of HCC patients.

Variable	Number with percentage and median with interquartile range	
	Total patients (n = 58)	Patients with peripheral blood sample (n = 43)
Gender		
Female	6 (10.3%)	5 (11.6%)
Male	52 (89.7%)	38 (88.4%)
Age (years)	57 (49–64)	59 (54–66)
Cirrhosis		
No	40 (69.0%)	28 (65.1%)
Yes	18 (31.0%)	15 (34.9%)
ALT (U/L)	24.0 (18.0–32.8)	24.0 (18.0–31.5)
AFP (ng/ml)	15.1 (3.8–878.5)	15.1 (3.7–1112.3)
ALBI score		
I	46 (79.3%)	35 (81.4%)
II	12 (20.7%)	8 (18.6%)
III	0 (0%)	0 (0%)
HBsAg		
Negative	10 (17.2%)	8 (18.6%)
Positive	48 (82.8%)	35 (81.4%)
HBV DNA		
Negative	33 (56.9%)	29 (67.4%)
Positive	23 (39.7%)	14 (32.6%)
Unknown	2 (3.4%)	0 (0%)
Type of HCC		
Primary	36 (62.1%)	24 (55.8%)
Recurrence	22 (37.9%)	19 (44.2%)
Number of lesions		
Single	43 (74.1%)	31 (72.1%)
Multiple	15 (25.9%)	12 (27.9%)
Tumor size (cm)	5.5 (3.1–8.0)	4.5 (2.9–6.5)
Tumor (T) stage		
1a	7 (12.1%)	7 (16.3%)
1b	33 (56.9%)	22 (51.2%)
2	8 (13.8%)	7 (16.3%)
3	9 (15.5%)	6 (13.9%)
4	1 (1.7%)	1 (2.3%)
Lymphoid nodal (N) status		
N0	58 (100%)	43 (100%)
N1	0 (0%)	0 (0%)
Distant metastasis (M) status		
M0	56 (96.6%)	41 (95.3%)
M1	2 (3.4%)	2 (4.7%)
TNM stage		
IA	7 (12.1%)	7 (16.3%)
IB	33 (56.9%)	22 (51.2%)
II	6 (10.3%)	5 (11.6%)
IIIA	9 (15.5%)	6 (13.9%)
IIIB	1 (1.7%)	1 (2.3%)
IVA	0 (0%)	0 (0%)
IVB	2 (3.4%)	2 (4.7%)
Tumor differentiation		
Well	0 (0%)	0 (0%)
Moderate	24 (41.4%)	18 (41.9%)
Poor	34 (58.6%)	25 (58.1%)

ALT, alanine aminotransferase; AFP, alpha-fetoprotein; ALBI, albumin–bilirubin; HBsAg, hepatitis B surface antigen; HBV, hepatitis B virus; HCC, hepatocellular carcinoma.

### Various TCRβ Repertoires Among Different Tissue Origins

**Figure 2:**
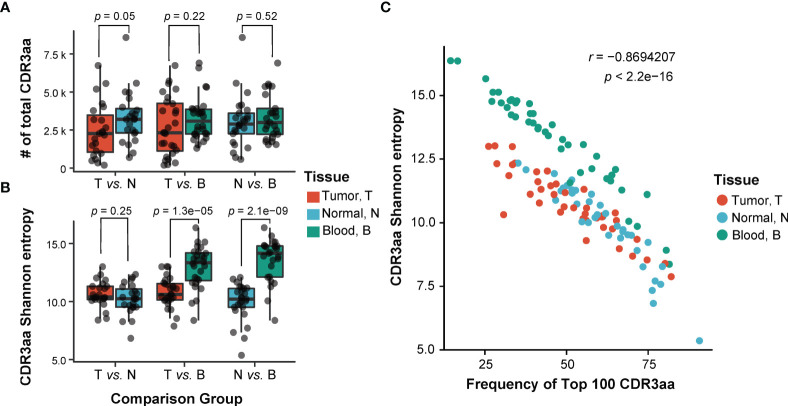
Comparison of TCRβ CDR3 characteristic among three different sites. **(A, B)** Comparison of the number **(A)** and the diversity **(B)** of TCRβ CDR3 amino acid sequences among three tissues. T, tumor; N, adjacent non-tumor tissues; B, peripheral blood. *p*-Values shown are according to paired t-test. **(C)** The correlation of the diversity of TCRβ CDR3 and abundance of top 100 CDR3. *p*-Values shown are according to t-test. TCR, T-cell receptor.

As part of the TCR variable chain, CDR3 is crucial for T-cell recognition of antigens, and it directly reflects the T-cell immune response status. To investigate differences of TCRβ repertoires across tumors, adjacent non-tumor tissues, and peripheral blood, we compared the number (**Figure 2A**) and the diversity (**Figure 2B**) of CDR3 aa among these tissues. There was no significant difference in the number of CDR3 among three types of tissues (T vs. N, *p* = 0.05, T vs. B, *p* = 0.22, N vs. B, *p* = 0.52; paired t-test; **Figure 2A**). Shannon’s entropy was used to measure the CDR3 diversity (38). A higher value of Shannon’s entropy indicates a higher diversity, which is also negatively correlated with the abundance of top 100 CDR3 (*r* = −0.869, *p* < 2.2e−16; t-test; **Figure 2C**). The CDR3 diversity was significantly higher in peripheral blood than in the other two types of tissues, whereas it was similar between tumor and non-tumor adjacent tissues (T vs. N, *p* = 0.25, T vs. B, *p* = 1.3e−05, N vs. B, *p* = 2.1e−09; paired t-test; **Figure 2B**). The result implied a higher TCR diversity in peripheral blood. When combined with the results of **Figures 2A**, **B**, we can speculate the existence of expanded TCR clones in both non-tumor and tumor tissues.

### The TCRβ CDR3 Heterogeneity in Hepatocellular Carcinoma Patients

**Figure 3:**
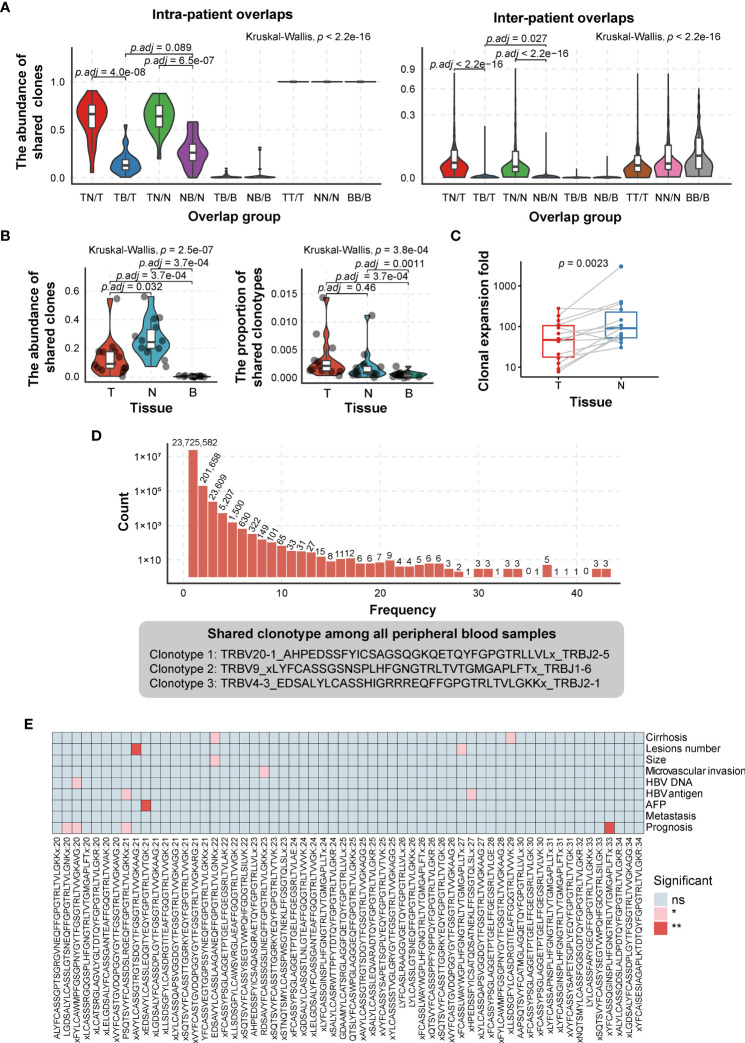
Comparison of TCRβ CDR3 heterogeneity in HCC patients. **(A)** Comparison of the abundance of shared clonotypes overlaps between any two tissues from the same patient (left graph) or between any two patients (right graph). **(B)** Comparison of the abundance (left graph) and types (right graph) of shared clonotypes among three tissues in a single tissue. T, tumor; N, adjacent non-tumor tissues; B, peripheral blood. TN, the shared clones/clonotypes overlapped between T and N; TB, the shared clones/clonotypes overlapped between T and B; NB, the shared clones/clonotypes overlapped between N and B; TNB, the shared clones/clonotypes overlapped among T, N, and B. *p*-Values are shown in panels A and B according to Kruskal–Wallis (for multiple groups’ comparison) and Wilcoxon test (for two groups’ comparison). *p*-Value adjusted by Bonferroni correction. **(C)** The degree of clonal expansion in adjacent non-tumor tissues, which was calculated on the basis of the ratio of the proportion of TNB shared clones in T/N to in B. It represents the clonal expansion rate of “TNB shared clones” in T/N compared to B. T, tumor; N, adjacent non-tumor tissues; B, peripheral blood. TNB, the shared clones overlapped among T, N, and B. *p*-Values are shown in panel C according to paired Wilcoxon test. **(D)** The distribution of shared CDR3 clonotypes in peripheral blood samples (top) and shared clonotypes among all 43 peripheral blood samples (bottom). **(E)** The association of the clonotypes, which are present in 20–34 (47%–80% in 43 samples) peripheral blood samples and patient clinical phenotype (considering the sample size of “without group,” the clonotypes, which are present in no more than 34 peripheral blood samples, are selected). ns, no significance, *p* > 0.05; *p < 0.05; **p < 0.01. TCR, T-cell receptor; HCC, hepatocellular carcinoma.

To investigate the intra- and inter-patient heterogeneity of the TCRβ CDR3 repertoire, the abundance of shared clonotypes was calculated between any two types of tissues from one patient or between any two patients. We observed that the abundance of shared clonotypes abundance between any two tissues within a patient was significantly higher than that between different patients (**Figure 3A**), revealing higher heterogeneity among patients. The tumor and adjacent non-tumor tissues shared higher fraction of clonotypes abundance than either of them shared with peripheral blood (Kruskal–Wallis test, *p* < 2.2e−16; the mean percentage of TN/T vs. TB/T = 0.608 ± 0.203 vs. 0.157 ± 0.117, *p* = 4.0e−08; TN/N vs. NB/N = 0.634 ± 0.162 vs. 0.274 ± 0.161, *p* = 6.5e−07; Wilcoxon test, *p*-value adjusted by Bonferroni correction; **Figure 3A**, left graph). This suggested that the majority of tumor-infiltrating T cells were recruited from adjacent non-tumor tissues rather than directly from peripheral blood. To some extent, the results calculated by TCR sequencing data in previous tumor environment studies (39, 40) were similar to our results can also support our point (**Supplementary Figures 2A, B** **)**. In addition, the clones shared between peripheral blood and adjacent non-tumor tissues were not significantly different from those shared between peripheral blood and tumors (Kruskal–Wallis test, *p* < 2.2e−16; the mean percentage of NB/N vs. TB/T = 0.274 ± 0.161 vs. 0.157 ± 0.117, *p* = 0.089; Wilcoxon test, *p*-value adjusted by Bonferroni correction; **Figure 3A**, left graph).

We further compared the proportion of shared clones and clonotypes among three tissues (TNB) in a single tissue. The proportion of shared clones means the abundance of shared clonotypes in each tissue, which is the ratio of reads of shared clonotypes to the total reads. The proportion of shared clonotypes refers to the ratio of the number of shared clonotypes to the number of all clone types. The results showed that the proportion of shared clones was the highest in adjacent non-tumor tissues, lower in the tumor, and the lowest in peripheral blood (the mean proportion of TNB/N = 0.266 ± 0.130, TNB/T = 0.135 ± 0.134, TNB/B = 3.68e−03 ± 3.00e−03, TNB/N vs. TNB/T, *p* = 0.032; TNB/T vs. TNB/B, *p* = 3.7e−04; paired Wilcoxon test, *p*-value adjusted by Bonferroni correction; **Figure 3B**, left graph). However, for the proportion of shared clonotypes, there was no difference between tumor and adjacent non-tumor tissues, while both tumor and adjacent non-tumor tissues had a higher proportion than peripheral blood (the mean proportion of TNB/T = 3.37e−03 ± 3.72 e−03, TNB/N = 2.11e−03 ± 2.81e−03, TNB/B = 6.47e−04 ± 5.11e−04, TNB/T vs. TNB/N, *p* = 0.46; TNB/N vs. TNB/B, *p* = 1.1e−03; TNB/T vs. TNB/B, *p* = 3.7e−04; paired Wilcoxon test; **Figure 3B**, right graph). The degree of clonal expansion in adjacent non-tumor tissues was significantly higher than that in tumoral tissues (340.03 vs. 76.94, *p* = 2.3e−03; paired Wilcoxon test) (**Figure 3C**), which implicated that the clonal expansion of tumor-infiltrating T cells was compromised in the tumoral immunosuppressive microenvironment.

Despite the obvious heterogeneity of CDR3 clonotypes among patients, we found three shared clones in all 43 peripheral blood samples (**Figure 3D**). The shared clonotypes are TRBV20-1_AHPEDSSFYICSAGSQGKQETQYFGPGTRLLVLx_TRBJ2-5, TRBV9_xLYFCASSGSNSPLHFGNGTRLTVTGMGAPLFTx_TRBJ1-6, and TRBV4-3_EDSALYLCASSHIGRRREQFFGPGTRLTVLGKKx_TRBJ2-1, with an average fraction of 0.558%, 0.407%, and 0.168%, respectively (**Figure 3D**). However, we did not find any annotation for peptides or antigens recognized by these TCRs from databases or published papers. Considering significant clonotypes are not necessarily present simultaneously in all peripheral blood samples, we selected clonotypes that present in most peripheral blood samples and grouped them into with or without an identical clonotype. Then we used the chi-square test to test the association between these clonotypes with patient clinical parameters. The result shows that several clonotypes are significantly associated with HCC prognosis, HBV DNA/antigen presence, cirrhosis, etc. (**Figure 3E**).

### TCRβ VJ Characteristics in Tumors, Adjacent Non-Tumor Tissues, and Peripheral Blood

**Figure 4:**
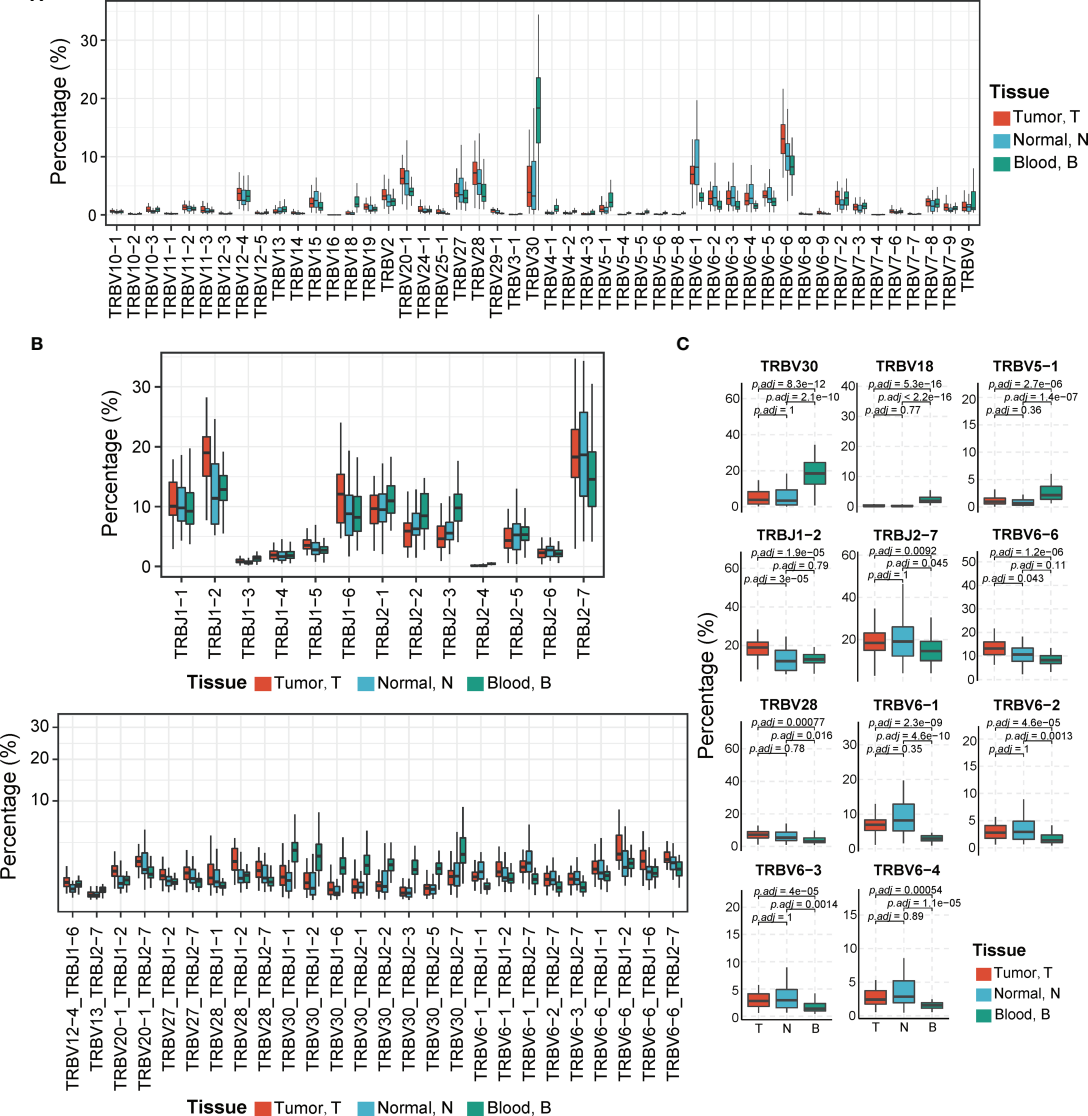
Comparison of TCR Vβ, Jβ, and Vβ–Jβ paired gene usage in tumor, adjacent non-tumor tissues, and peripheral blood. **(A)** The usage abundances of 48 Vβ genes in three tissues. T, tumor; N, adjacent non-tumor tissues; B, peripheral blood. **(B)** The usage abundances of 16 Jβ genes in three tissues. **(C)** Vβ or Jβ genes have significant differential usage abundances in different tissues. *p*-Values are shown in the figure according to Kruskal–Wallis (for multiple groups’ comparison) and Wilcoxon test (for two groups’ comparison). *p*-Value adjusted by Bonferroni correction. **(D)** The usage abundances of 26 TCR Vβ–Jβ paired genes that have high abundance in three tissues (mean abundance >1%, *p*-Values <0.01, Kruskal–Wallis test). TCR, T-cell receptor.

To profile the landscape of TCRβ V and J genes, we calculated the usage abundance of TCRβ V and J gene segments for every sample in all three tissue types. In abundance heatmap result (**Supplementary Figure 3A**), we can find that the usage pattern of Vβ genes in peripheral blood such as TRBV18, etc., are seemingly distinguished from tumor and adjacent non-tumor tissues. In correlation coefficient analysis (**Supplementary Figure 3B**), the majority of correlation coefficients higher than 0.6 show the overall similarity across different tissues. However, we can also find that the usage pattern of Vβ genes and VJ pairings in peripheral blood is seemingly distinguished from tumor and adjacent non-tumor tissues. In general, usage patterns of Vβ genes in peripheral blood were a little distinguished from those in tumor and adjacent non-tumor tissues, while the usage patterns of Jβ genes in different tissues were similar. In the respective five most abundant Vβ genes of three tissues, we found that there were four overlapped ones, including TRBV6-6 (average ratio in T, N, B: from 9.07% ± 4.94% to 14.25% ± 6.48%), TRBV30 (average ratio in T, N, B: from 5.46% ± 5.25% to 18.99% ± 9.63%), TRBV28 (average ratio in T, N, B: from 4.94% ± 4.83% to 8.75% ± 11.01%), and TRBV20-1 (average ratio in T, N, B: from 4.75% ± 3.87% to 6.89% ± 2.97%). In the respective five most abundant Jβ genes of three tissues, the Jβ gene TRBJ2-7 (average ratio in T, N, B: from 14.95% ± 6.15% to 20.34% ± 10.14%) ranked the highest in all tissues. Other overlapped Jβ genes were TRBJ1-2 (average ratio in T, N, B: from 12.87% ± 6.77% to 19.33% ± 6.42%) and TRBJ2-1 (average ratio in T, N, B: from 10.21% ± 4.40% to 11.90% ± 4.85%; **Figures 4A**, **B**). TRBV20-1, TRBJ2-7, and TRBJ2-1 have also been reported as the most common abundant gene segments in previous studies of HBV-associated HCC, pancreatic cancer, and nasopharyngeal carcinoma, while TRBV6-6 has also been found as the frequent gene segment in another study of HCC (23, 26, 28, 41).

Some V and J gene segments demonstrated significantly different usage abundances in different tissues (all *p* < 0.05, Kruskal–Wallis test) (**Figure 4C**). For example, TRBV30, TRBV18, and TRBV5-1 exhibited significantly higher usage abundances in peripheral blood than those in tumoral and peritumoral tissues (all adjusted *p* < 0.05, Wilcoxon test, *p*-value adjusted by Bonferroni correction). It is reported that TRBV30 has low usage abundance in healthy blood donors and some diseases, including acute myocardial infarction (AMI) or colorectal cancer (CRC) (42–45) but exhibited high usage abundances in the peripheral blood of patients infected with H5N6 influenza virus and patients with systemic lupus erythematosus (37, 46). TRBV18 was detected more abundantly in tumor tissues than in adjacent non-tumor tissues of lung cancer (47). TRBJ1-2 was used more abundantly in tumors than in adjacent non-tumor tissues (adjusted *p* = 3e−05, Wilcoxon test, *p*-value adjusted by the Bonferroni correction). The tissue distribution pattern of TRBJ1-2 was consistent with that in the previous study of HBV-associated HCC (27). In addition, TRBV6-1/2/3/4, TRBV28, and TRBJ2-7 exhibited significantly higher abundances in tumor and adjacent non-tumor tissues than in peripheral blood (all adjusted *p* < 0.05, Wilcoxon test, *p*-value adjusted by the Bonferroni correction). Moreover, TRBV6-6 showed the highest usage in tumor tissues, lower in adjacent non-tumor tissues, and the lowest in peripheral blood, which implied that the expansion of this TCRβ V gene-related clones may be due to stimulation of tumor antigens (adjusted *p* < 0.05, Wilcoxon test, *p*-value adjusted by the Bonferroni correction).

We also compared the usage pattern of TCRβ VJ pairings among three tissues. There were 26 dominant VJ pairings (mean abundance among three tissues >1%) showing significant differences in their usage abundances among three tissues (*p* < 0.01, Kruskal–Wallis test; **Figure 4D**). Among them, VJ pairings such as TRBV20-1_TRBJ1-2, TRBV28_TRBJ1-2, and TRBV6-6_TRBJ1-2 were significantly enriched in the tumor, whereas other VJ pairings such as TRBV30_TRBJ1-1, TRBV30_TRBJ1-2, and TRBV30_TRBJ2-7 exhibited higher abundance in peripheral blood.

### Correlation Between the T-Cell Receptor Repertoire and Clinical Characteristics

Later on, we assessed the association between the TCRβ CDR3 features and HCC clinical characteristics, including tumor size, microvascular invasion (MVI), and the AFP concentration in the blood. TCR diversities of three tissues showed no significant association with any clinical features (**Supplementary Figure 4A**). However, the proportion of shared clones among different tissue types showed association with some clinicopathological characteristics (**Supplementary Figures 4B, C** **)**. Patients with a lower risk of postoperative recurrence tended to have a higher proportion of blood-peritumoral tissues shared clones in blood TCR repertoire (NB/B) (*p* < 0.05, Wilcoxon test; **Supplementary Figure 4B**). A higher proportion of blood-peritumoral tissues shared clones in peritumoral TCR repertoire (NB/N) was correlated with non-MVI, tumor size <5 cm, and negative HBV-DNA load (all *p* < 0.05, Wilcoxon test; **Supplementary Figure 4B** **)**. Moreover, patients without MVI exhibited a higher proportion of tumor–peritumoral tissues shared clones in peritumoral TCR repertoire (TN/N) than those with MVI (*p* < 0.05, Wilcoxon test; **Supplementary Figure 4B**). Furthermore, non-relapse patients tended to have a higher proportion of three tissues shared clones in tumoral TCR repertoire (TNB/T) (*p* < 0.05, Wilcoxon test; **Supplementary Figure 4C**).

### Distinct TCRβ VJ Pairing Usage of Peripheral Blood in HCC Patients

**Figure 5:**
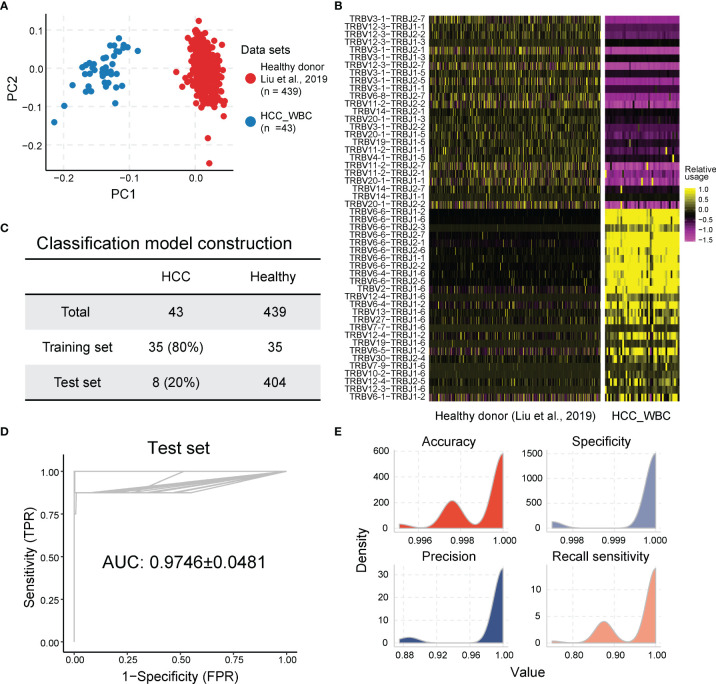
The usage of TCR Vβ–Jβ paired genes are the potential biomarkers for HCC diagnosis. **(A)** Principal component analysis based on TCR Vβ–Jβ paired gene usage profile. **(B)** Heatmap of different TCR Vβ–Jβ paired gene usage patterns between HCC patients and healthy donors. The heatmap bar indicates the scaled abundance of TCR Vβ–Jβ paired genes. **(C)** The cohorts of peripheral blood samples from HCC patients and healthy donors were used in LASSO model; 80% (n = 35) of the HCC cohort (n = 43) were randomly selected as the training set and the remaining 20% (n = 8) as the validation set. Healthy donors included in each set were also randomly selected from healthy cohort (n = 439), and their number was the same as the number of HCC patients. **(D, E)** Performance of diagnosis LASSO model repeated 100 times in training cohort with AUC of 0.9746 ± 0.0481 (average of 100 repeats; left graph) and sensitivity of 0.9675 ± 0.0603, specificity of 0.9998 ± 0.0007, accuracy of 0.9992 ± 0.0013, and precision of 0.9908 ± 0.0311 (average of 100 repeats; right graph). AUC, under the curve; TPR, true-positive rate; FPR, false-positive rate. The ROC and average AUC of 100 repeats using LASSO models are shown in the left panel. The density for accuracy, specificity, precision, and sensitivity of the prediction results from 100 repeats using LASSO model is shown in the right panel. TCR, T-cell receptor; HCC, hepatocellular carcinoma; LASSO, least absolute shrinkage and selection operator; AUC, area under the curve; ROC, receiver operating characteristic.

Considering that the TCR repertoire in peripheral blood is a systemic reflection of the immune response of tumors (48), we postulated that the features of TCRβ VJ pairings in the peripheral blood may potentially serve as a diagnostic biomarker. We first analyzed the data of peripheral TCR repertoires from 439 healthy controls from Liu’s published dataset (37) and 43 HCC patients in the cohort. PCA based on TCRβ VJ pairing usage profile showed that HCC patients were distinctly different from healthy people, revealing a unique TCR repertoire in HCC patients (**Figure 5A**). The differential analysis identified 90 TCRβ VJ pairings showing significantly different abundances between HCC patients and healthy donors. Among them, 65 TCRβ VJ pairings were more abundant in healthy donors, including TRBV3-1_TRBJ2-1 (healthy vs. HCC = 0.314 ± 0.202 vs. 0.015 ± 0.013) and TRBV12-3_TRBJ2-7 (healthy vs. HCC = 0.270 ± 0.173 vs. 0.019 ± 0.018). On the other hand, 25 TCRβ VJ pairings were enriched in HCC patients, including TRBV6-6_TRBJ2-1 (HCC vs. healthy = 0.755 ± 0.314 vs. 0.048 ± 0.047) and TRBV27_TRBJ1-6 (HCC vs. healthy = 0.325 ± 0.509 vs. 0.085 ± 0.010) (**Figure 5B**).

To establish a robust model for using the peripheral TCRβ VJ pairings as a biomarker for HCC diagnosis, peripheral TCRβ VJ pairings of 43 HCC samples and 439 healthy controls were acquired as the training cohort (80%) and testing cohort (20%) by using random partitioning for HCC diagnosis model construction (experimental section and **Figure 5C**). The baseline characteristics of the training cohort and test cohort of HCC patients are shown in **Table 1**. We used the LASSO regression for TCRβ VJ pairing for logistic regression to develop a model for HCC rapid diagnosis. HCC patients can be differentiated from healthy people with an AUC of 0.9746 ± 0.0481 (sensitivity = 0.9675 ± 0.0603, specificity = 0.9998 ± 0.0007, average of 100 repeats) in the testing cohort (**Figures 5D** **, E**). The results above support that TCRβ VJ pairing usage in peripheral blood has strong discriminability to identify HCC patients who were selected in each repeat and may be a highly promising biomarker for HCC diagnosis.

## Discussion

T cells are widely known to participate in the tumor-immune interaction and play an important role in anticancer activities. As T cells recognize tumor antigens *via* a diverse range of TCRs, by analyzing features of the TCR repertoire containing TCRβ CDR3 and VJ genes, we may deepen our understanding of cancer progression and immune response in the tumor microenvironment. Several previous studies showed such TCR repertoire features and their diagnosis or prognosis value in some tumor types (23, 49). However, a systemic characterization of the TCR repertoire in peripheral blood of patients with HCC and its clinical importance is lacking in the study. Our study comprehensively delineates the features of TCR repertoires in the tumor, adjacent non-tumor tissues, and peripheral blood from HCC patients using high-throughput TCR sequencing. We observe a significantly higher TCR diversity (TCRβ CDR3 level) in peripheral blood and a similar TCR diversity between tumor and adjacent non-tumor tissues. However, there is no obvious difference in the TCRβ CDR3 aa number among these tissues. These results imply a T-cell expansion in both the tumor and adjacent non-tumor tissues. Moreover, the analysis of shared TCRβ CDR3 indicates that the majority of clonotypes shared by tumors and adjacent non-tumor tissues are clonally expanded. Although the usage patterns of Vβ and Jβ genes show overall similarity among three tissues, we detect some gene segments with significantly different usage abundances, such as TRBV30, TRBV18, TRBJ1-2, and TRBJ1-6. Interestingly, several Vβ gene segments, such as TRBV6-6, exhibit a gradually increased usage from peripheral blood to adjacent non-tumor tissues to the tumor, which indicates that the neoantigen recognition process within the tumor leads to the expansion of specific T-cell clones.

For resectable HCC, recurrence and metastasis are the main causes of poor prognosis (6). Early recurrences, defined as relapse within 2 years of primary HCC resection, are frequently attributed to intrahepatic micrometastasis of HCC (50). Cancer cell spreading *via* the microvascular systems in peritumoral liver tissues, which is defined as MVI, has been generally considered to be the main mechanism for intrahepatic micrometastasis in HCC (51). Emerging evidence has implicated an association of micrometastasis with compromised immune surveillance function (52). However, the underlying mechanism by which immune cells influence the development of MVI in HCC is largely unknown. In the current study, we demonstrate that MVI-positive patients have a significantly lower proportion of tumor–peritumoral tissues (TN/N) shared clones in the peritumoral MVI repertoire than those without detected MVI. This result suggests that there are fewer memory T cells that recognize tumor antigens in the peritumoral liver tissues of patients with MVI, which will lead to ineffective immune monitoring of disseminated cancer cells. Our data support the idea that infiltration by tissue-resident memory T cells in peritumoral liver tissues plays a critical role in the immune surveillance on the development of MVI in HCC.

T cells can recognize and eliminate nascent transformed cells, but this response is transient. Most of the tumor-associated T cells that recognize and kill cancer cells are eliminated from the circulation along with the effective clearance of precancerous and/or cancerous cells (53). During tumorigenesis, the TCR repertoire would undergo constant turnover driven by the emergence and clearance of sporadic cancer cells (54). When cancer cells manage to survive immune destruction and enter “equilibrium” or “escape” states, tumor-associated T cells would be constantly generated (55). Thus, the accumulated tumor-specific TCR repertoire features can be detected and reflect the presence of tumor cells (56, 57). Consistent with this, cancer-associated T cells have been detected in the blood of patients with early-stage CRC and melanoma (58, 59). Moreover, a recent study reported a DeepCAT model that was trained for non-invasive cancer detection by predicting cancer-associated TCRs in peripheral blood (60). The prognostic significance of blood TCR repertoire in patients with advanced lung cancer and melanoma has been reported (48, 61). However, whether peripheral TCR repertoire can serve as an independent diagnostic or prognostic biomarker for HCC had not been elucidated. In the current study, we establish a panel of TCRβ VJ pairings in the peripheral blood that is capable of accurately discriminating HCC patients from healthy individuals with sensitivity of 0.9675 ± 0.0603 and specificity of 0.9998 ± 0.0007. Our data demonstrate the potential of peripheral TCR repertoire as an effective clinical diagnostic biomarker for HCC patients.

There are several limitations of this study. First, the data used in our study were from a single center, and the cohort size was relatively small. Second, this study lacked data from patients with hepatitis B and cirrhosis as a control. Therefore, a randomized, large-scale, and multicenter study is needed to further verify the clinical significance of TCR sequence in HCC, especially the guiding significance of diagnosis or prognosis of TCR repertoire in HCC.

In conclusion, by using high-throughput TCR sequencing, we have comprehensively characterized TCR repertoires in the tumor, adjacent non-tumor tissues, and peripheral blood from HCC patients, which provide a deeper understanding of the T-cell immune response in HCC patients. Moreover, our findings suggest that the circulating TCRβ VJ pairing usage pattern may be a clinically useful diagnostic biomarker for HCC patients.

## Data Availability Statement

The datasets presented in this study can be found in online repositories, which deposited into the CNGB Sequence Archive (CNSA) (62) of the China National GeneBank DataBase (CNGBdb, https://db.cngb.org/cnsa/) (63) with accession number CNP0002126.

## Ethics Statement

The studies involving human participants were reviewed and approved by the Institutional Ethics Committee of the Zhongshan Hospital, Fudan University. The patients/participants provided their written informed consent to participate in this study.

## Author Contributions

ZW, YZ, ZZ, and KZ contributed equally to this work. LW and YS: study conceptualization and manuscript revision. ZW, YZ, and ZZ: paper writing. ZW: performing experiments. YZ, ZH, and HY: data analysis and figures. ZZ and KZ: clinical data collection. LL, SL, HMY, JZ, and JF: study progress supervision. All authors listed have made a substantial, direct, and intellectual contribution to the work and approved it for publication.

## Funding

This work was supported by the Guangdong-Hong Kong Joint Laboratory on Immunological and Genetic Kidney Diseases (019B121205005) Shenzhen Key Laboratory of Single-Cell Omics (ZDSYS20190902093613831), the National Natural Science Foundation of China (82073222), Shanghai Municipal Health Commission Collaborative Innovation Cluster Project 2019CXJQ02), and the Rising-Star Program from the Shanghai Science and Technology Commission (19QA1402000).

## Conflict of Interest

The authors declare that the research was conducted in the absence of any commercial or financial relationships that could be construed as a potential conflict of interest.

## Publisher’s Note

All claims expressed in this article are solely those of the authors and do not necessarily represent those of their affiliated organizations, or those of the publisher, the editors and the reviewers. Any product that may be evaluated in this article, or claim that may be made by its manufacturer, is not guaranteed or endorsed by the publisher.

## Abbreviations

HCC, hepatocellular carcinoma; TCR, T-cell receptor; TCRβ, T-cell receptor beta locus; CDR3, complementarity-determining region 3; AFP, alpha-fetoprotein; MVI, microvascular invasion.
