# Supplementary material for: Characteristics and Clinical Significance of T-Cell Receptor Repertoire in Hepatocellular Carcinoma
Source: Front Immunol. 2022 Mar 16;13:847263. doi: 10.3389/fimmu.2022.847263 (PMC8965762; doi:10.3389/fimmu.2022.847263)
Supplement: Supplementary file 1 [file DataSheet_1.pdf]

Supplementary Material

Supplementary Figure legends

SupFig. 1. Profiling the TCR repertoire sequencing data in each sample. The number of clean reads, CDR3 amino acid clonotypes and CDR3 nucleotide clonotypes in 40 tumor, 37 adjacent normal tissues and 43 peripheral blood samples from 58 HCC patients. T, tumor; N, adjacent normal tissue; B, peripheral blood.

SupFig. 2. Comparison of the proportion of shared clones in non-tumor and peripheral blood. (A) Comparison of TN/T, TB/T (left graph) and TNB/T, TNB/N (right graph). The data of 12 patients with non-small-cell lung cancer was from Zhang, et al. published in 2018. (B) Comparison of TN/T, TB/T (left graph) and TNB/T, TNB/N (right graph). The data of 4 patients with liver cancer was from Zhang, et al. published in 2017. The non-significant tendency may be due to the small sample size. T, tumor; N, adjacent nontumor tissue; B, peripheral blood. TN, the shared clones overlapped between T and N; TB, the shared clones or overlapped between T and B; TNB, the shared clones overlapped among T, N, and B. P-values are shown in panels A and B according to paired Wilcoxon test.

SupFig. 3. Comparison of the usage patterns of TCR Vβ and Jβ genes in tumor, adjacent normal tissue and peripheral blood. (A)The heatmap of TCR Vβ and Jβ genes usage profile in tumor, adjacent normal tissue and peripheral blood. The color indicated the log2 scaled abundance of TCR Vβ and Jβ genes in each sample. (B) The correlation coefficient analysis of TCR Vβ and Jβ genes usage profile in tumor, adjacent normal tissue and peripheral blood. The color indicated the correlation coefficient of TCR Vβ and Jβ genes in each sample.

SupFig. 4. The association between the TCRβ CDR3 features and HCC clinical phenotypes. (A) The relationship between HCC clinical phenotypes and clonotypes diversity of tumor, adjacent normal tissues and peripheral blood samples. (B) The relationship between HCC clinical phenotypes and the proportion of clones overlapped between tissue pairs in the indicated tissue. T, tumor; N, adjacent normal tissue; B, peripheral blood; TN, the shared clones overlaps of T and N; TB, the shared clones overlaps of T and B; NB, the shared clones overlaps of N and B. (C) The relationship between HCC clinical phenotypes and the proportion of clones overlapped among three tissues in the indicated tissue. TNB, the shared clones overlaps of T, N, and B. * indicates p-value < 0.05, Wilcoxon test.

Supplemental Figure 1

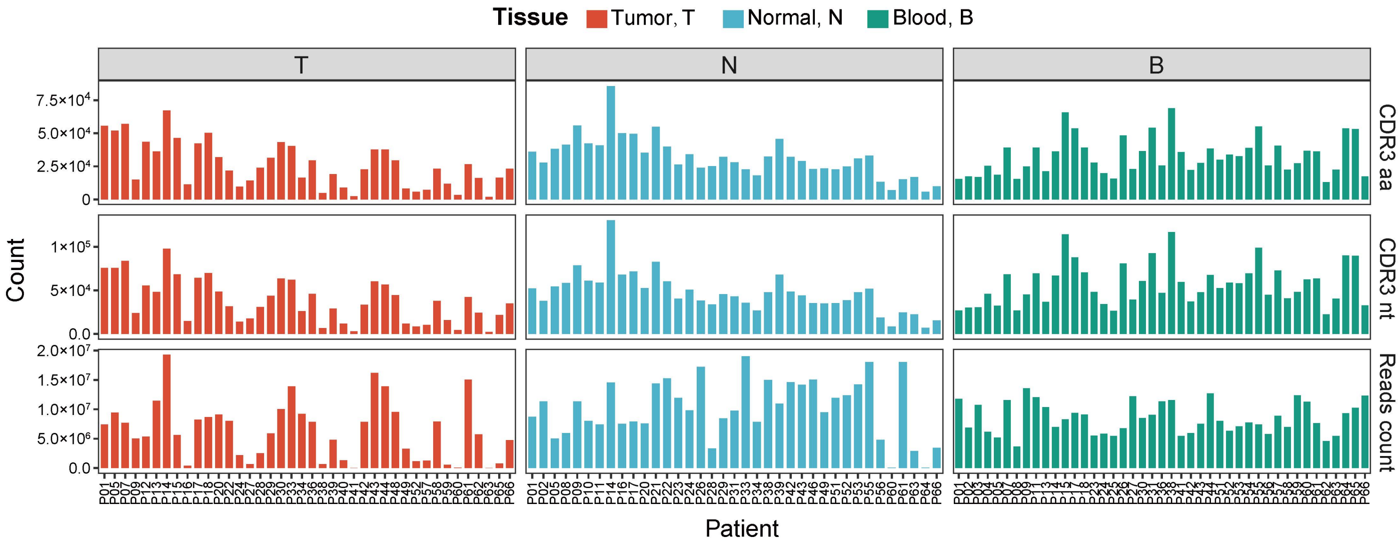

Supplemental Figure 2

A

B

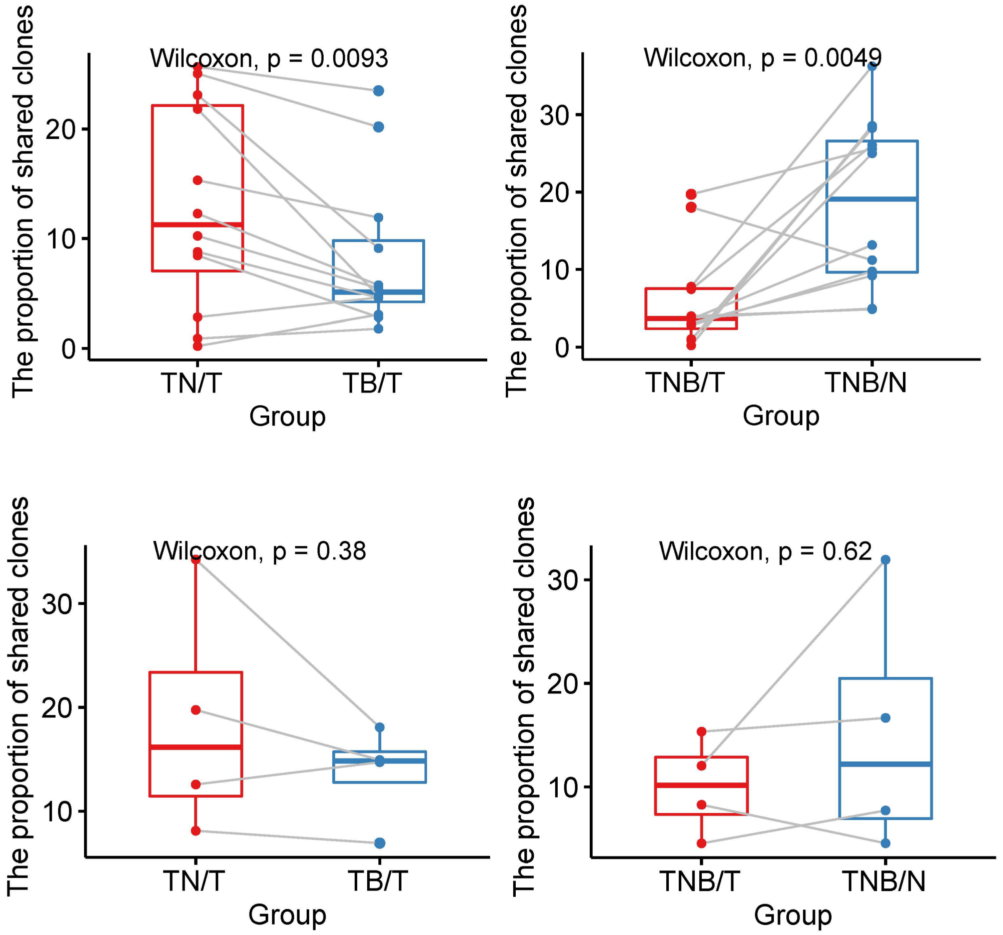

Supplemental Figure 3

A

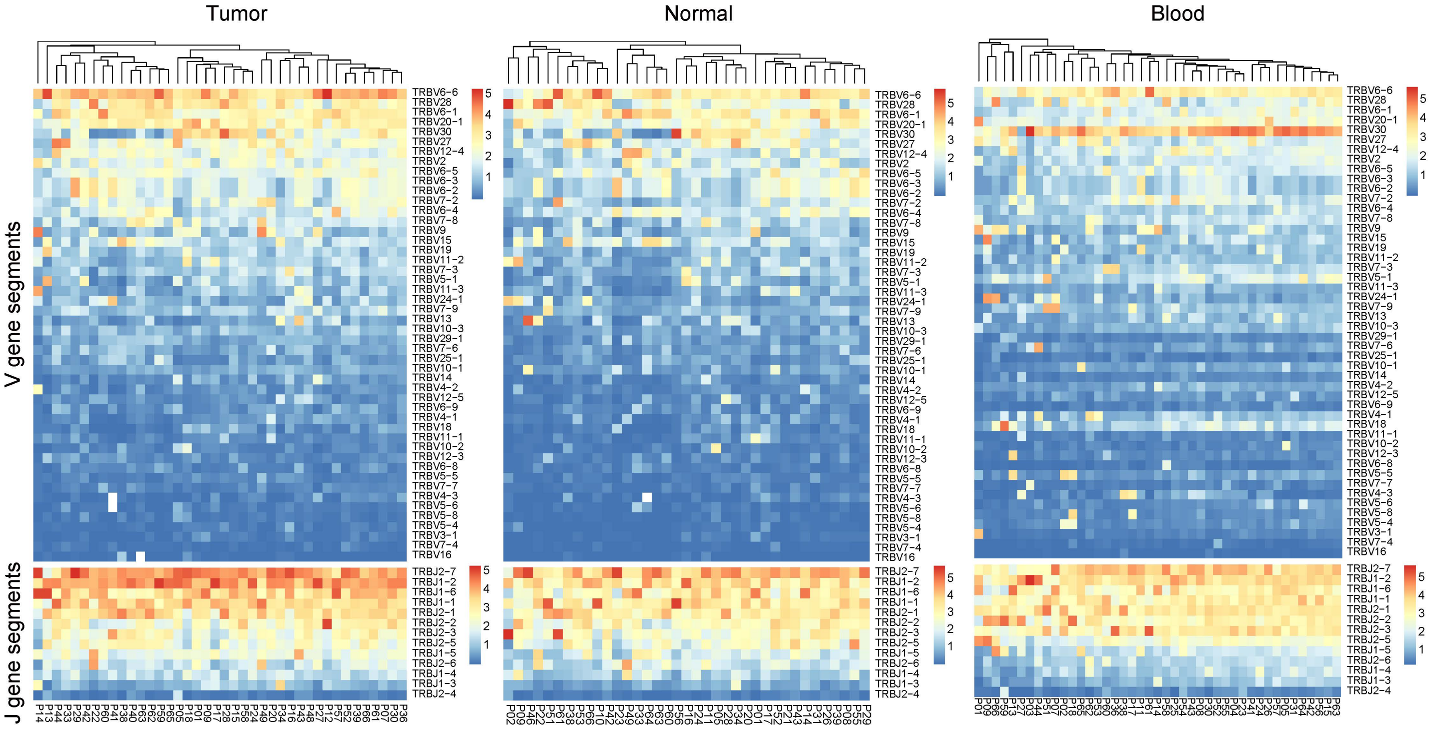

B

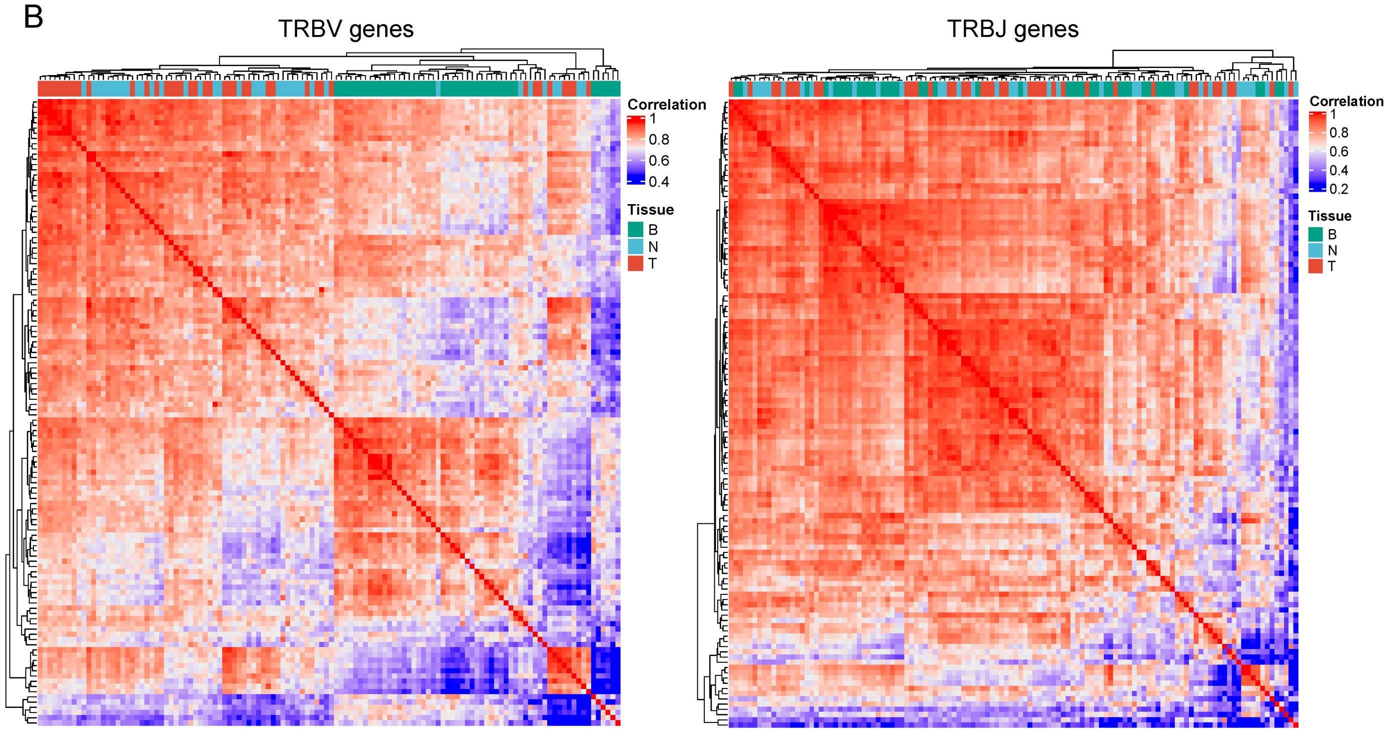

Supplemental Figure 4

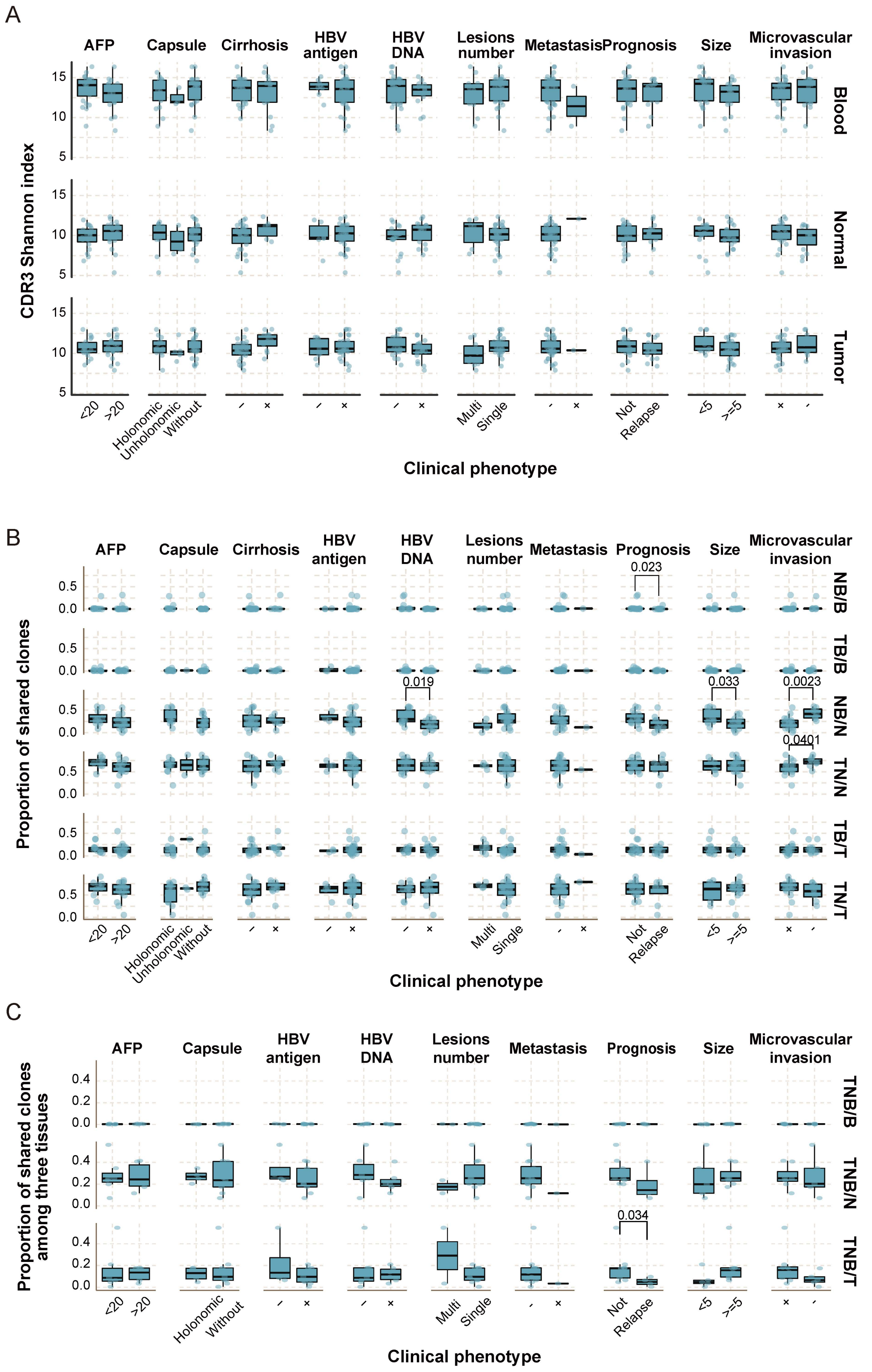

Supplementary Table 1. The detailed sequencing data information

	Patient	Tissue	Clean_reads	cdr3_nt	cdr3_aa	Shannon cdr3_nt	Shannon cdr3_aa	Shannon _V	Shannon _J
B01_T	P01	T	10018161	75515	55592	10.19	9.82	4.42	3.23
B01_N	P01	N	10316456	52227	36024	8.27	7.83	4.32	2.96
B01_B	P01	B	15791036	27030	15332	5.74	5.46	3.54	2.85
B02_N	P02	N	12738785	37988	27753	4.12	3.75	2.51	2.2
B02_B	P02	B	11702050	30322	17413	8.99	8.45	4.39	3.2
B03_B	P03	B	14952375	30683	16840	6.13	5.39	3.17	2.45
B04_B	P04	B	10790606	46139	25374	11.14	10.61	4.15	3.23
B05_T	P05	T	11363705	75550	51908	8.58	8.63	4.38	2.99
B05_N	P05	N	6582404	54626	38079	10.88	10.51	4.65	3.19
B05_B	P05	B	12527558	32366	18649	10.59	10.15	4.2	3.32
B07_T	P07	T	9803621	83763	57224	11.68	11.25	4.41	3.2
B07_B	P07	B	16978147	68049	39056	8.24	7.87	4.05	2.88
B08_N	P08	N	7539565	58500	41441	10.13	9.64	4.55	3.1
B08_B	P08	B	7815133	26924	15466	10.96	10.51	4.72	3.23
B09_T	P09	T	6853431	23939	14891	9.35	8.91	4.35	3.11
B09_N	P09	N	13450753	78432	55781	6.95	7.52	3.92	3
B09_B	P09	B	17927246	45486	24858	7.24	7.05	3.71	2.89
B10_N	P10	N	9554045	60965	42312	8.01	7.56	3.7	2.63
B11_N	P11	N	9194762	58898	40800	9.56	9.17	4.43	3.07
B11_B	P11	B	17287383	69197	39151	10.01	9.88	4.47	3.22
B12_T	P12	T	7660203	55705	43602	7.66	7.11	3.78	2.9
B13_T	P13	T	14217602	48156	36179	5.87	6.28	3.71	2.85
B13_B	P13	B	14207199	36750	21270	6.92	6.53	4.02	2.89
B14_T	P14	T	23508196	97623	67404	7.27	6.62	3.85	2.48
B14_N	P14	N	18542746	130596	85832	10.63	10.21	4.04	3.09
B14_B	P14	B	10128830	66656	36217	10.74	10.19	4.6	3.28
B15_T	P15	T	7217063	68236	46367	11.89	11.49	4.5	3.14
B15_B	P15	B	11839132	114314	65858	13.19	12.75	4.47	3.39
B16_T	P16	T	1243238	14631	11261	9.94	9.96	4.78	2.86
B16_N	P16	N	9623368	67660	50063	9.46	9.2	4.5	2.94
B17_T	P17	T	10085561	64231	42293	9.82	9.58	4.44	2.75
B17_N	P17	N	9647794	71505	49592	10.22	9.92	4.63	3.19
B17_B	P17	B	13122382	87673	53598	10.98	10.8	4.53	3.22
B18_T	P18	T	11424287	69552	50207	8.73	8.24	4.42	2.78
B18_B	P18	B	13043799	70419	39060	10.05	9.31	4.38	3.08
B20_T	P20	T	11251697	48637	31867	8.99	8.61	4.52	3.06
B20_N	P20	N	9508480	52768	35265	10.12	9.65	4.46	3.23
B21_N	P21	N	16835844	82475	54947	10.23	9.88	4.27	3.26
B22_T	P22	T	9190219	31711	21682	7.34	6.85	4.01	3.09
B22_N	P22	N	17675199	60033	39972	7.23	6.73	3.9	3.14
B23_N	P23	N	13861337	40456	26245	6.38	7.3	3.6	2.52
B23_B	P23	B	8069086	48270	27863	11.22	11.11	4.34	3.31
B24_T	P24	T	3403674	14043	9613	9.5	9.13	4.57	3.03
B24_N	P24	N	12319566	50815	33988	9.58	9.13	4.47	3.23
B24_B	P24	B	12412765	34351	19713	10.23	9.95	4.46	3.4
B25_B	P25	B	10121598	26668	15750	9.72	9.23	4.37	3.18
B26_N	P26	N	19936065	38484	23947	9.54	9.57	4.27	3.1
B26_B	P26	B	9543628	80625	48356	12.31	12.09	4.61	3.24
B27_T	P27	T	1708335	17664	14267	8.32	8.06	4.24	3

B27_B	P27	B	16688862	39308	22847	8.39	7.82	4.55	3.05
B28_T	P28	T	4470855	30882	24007	8.49	8.17	4.09	3
B28_N	P28	N	4610345	34077	25198	9.84	9.69	4.62	3.06
B29_T	P29	T	7564484	43810	31407	8.92	8.41	4.21	2.86
B29_N	P29	N	10567919	45521	32044	8.88	8.52	4.34	3
B30_T	P30	T	12187570	63483	43363	10.89	10.52	4.44	3.15
B30_B	P30	B	12466504	60634	36538	10.6	10.44	4.43	3.33
B31_N	P31	N	11836042	43018	28042	9.62	9.34	4.31	3.15
B31_B	P31	B	12299623	92418	54097	11.64	11.3	4.31	3.36
B33_T	P33	T	19872332	62030	40271	10.38	10.43	4.38	3.27
B33_N	P33	N	30977907	35691	22728	7.5	8.15	3.85	3.13
B34_T	P34	T	11622189	25978	16268	8.97	8.91	4.48	3.18
B34_N	P34	N	9348455	26749	18051	8.29	8.12	4.02	3.17
B36_T	P36	T	9550778	46072	29556	11.4	11.01	4.67	3.2
B36_B	P36	B	16510949	47254	25539	8.77	8.72	4.21	2.96
B38_T	P38	T	883546	6522	4829	9.08	8.82	4.26	2.99
B38_N	P38	N	19238224	48099	32374	8.55	8.29	4.07	3.14
B38_B	P38	B	15561193	116727	69012	11.99	11.67	4.36	3.21
B39_T	P39	T	6076759	28941	19167	11.36	10.97	4.67	3.14
B39_N	P39	N	13192071	67772	45778	11.27	10.88	4.6	3.18
B40_T	P40	T	1782456	11916	8922	9.95	9.57	4.44	3.11
B41_T	P41	T	19752	2740	2247	8.88	8.45	4.26	3.1
B41_B	P41	B	7783351	59326	35789	11.15	10.55	4.06	3.31
B42_T	P42	T	9955713	33508	22665	9.11	8.62	4.52	3.1
B42_N	P42	N	16753825	48565	32033	9.3	9.02	4.04	3.28
B42_B	P42	B	10966766	37069	22197	10.93	10.5	4.45	3.35
B43_T	P43	T	19138982	60046	37747	10.05	9.64	4.77	3.24
B43_N	P43	N	16353803	44393	28961	8.88	8.49	4.54	3.19
B43_B	P43	B	12915685	47797	27645	9.92	9.22	4.56	3.19
B44_T	P44	T	16521378	56903	37635	8.91	8.38	4.06	3.05
B44_B	P44	B	17538305	67396	38489	9.59	10.21	4.29	3.09
B46_N	P46	N	17392374	35529	22827	6.47	5.97	3.6	2.65
B48_T	P48	T	11469736	44569	29486	11.15	10.9	4.85	3.24
B49_T	P49	T	4711537	11926	8185	7.96	7.43	4.29	3.19
B49_N	P49	N	11290082	35117	23519	6.89	6.84	3.61	3.17
B51_N	P51	N	13464335	35193	22606	6.46	6.01	3.34	2.71
B51_B	P51	B	11070389	52592	29909	7.99	7.94	3.93	2.87
B52_T	P52	T	1954591	8342	5793	9.97	9.74	4.53	3.17
B52_N	P52	N	14434414	38793	24968	9.23	8.95	4.57	3.11
B52_B	P52	B	9501717	59154	33956	11.65	11.44	4.42	3.31
B53_N	P53	N	16697375	47918	30931	10.17	9.84	4.41	3.12
B53_B	P53	B	11340077	58211	32595	11.29	10.9	4.61	3.28
B54_B	P54	B	11876200	69297	38866	11.49	10.95	4.73	3.37
B55_N	P55	N	20622666	51869	33020	9.04	8.73	4.16	3.21
B55_B	P55	B	10696390	98866	55172	13.03	12.59	4.46	3.32
B56_N	P56	N	5435546	18828	13206	6.52	6.04	3.33	2.57
B56_B	P56	B	9756309	45154	25574	11.13	10.64	4.35	3.32
B57_T	P57	T	2013829	10324	7220	9.09	9.22	4.33	2.98
B57_B	P57	B	12580617	72787	40606	10.87	10.61	4.26	3.34
B58_T	P58	T	9616472	38022	23239	10.36	9.88	4.57	3.22
B58_B	P58	B	11303909	40769	22482	9.73	9.13	4.59	3.16
B59_T	P59	T	1223929	15636	11767	10.05	9.54	4.05	2.9
B59_B	P59	B	16183427	48133	27178	7.12	6.57	3.76	3

B60_T	P60	T	280329	4287	3369	9.26	8.92	4.23	3.1
B60_N	P60	N	108389	8281	6944	10.28	9.93	4.18	3.11
B60_B	P60	B	14347508	62187	36711	8.51	7.89	4.07	3.16
B61_T	P61	T	18321698	42521	26482	10.74	10.43	4.59	3.28
B61_N	P61	N	21454199	24551	15263	5.59	5.36	3.13	2.41
B61_B	P61	B	10524784	63243	36158	8.87	8.41	3.81	2.87
B62_T	P62	T	7520867	24424	16107	9.91	9.61	4.34	3.16
B62_B	P62	B	8994179	22495	13024	8.78	8.33	4.34	3.27
B63_T	P63	T	6559	2183	1889	10.28	10.01	4.59	3.18
B63_N	P63	N	3644373	22584	16871	9.35	8.94	3.82	3.03
B63_B	P63	B	10212323	40679	22499	11.48	11.03	4.57	3.36
B64_N	P64	N	144691	6835	5701	9.3	9.14	4.2	3.02
B64_B	P64	B	13142351	89782	53762	11.45	10.93	4.13	3.33
B65_T	P65	T	1055329	21898	16342	10.66	10.39	4.33	3.08
B65_B	P65	B	13463356	89714	53166	9.53	9.19	3.71	3.21
B66_T	P66	T	6026442	34974	23209	11.53	11.47	4.4	3.29
B66_N	P66	N	4313648	15502	9922	7.49	8.21	3.99	3.17
B66_B	P66	B	16530679	32694	17360	7.12	7.11	3.86	3.1
